# MMP9 Knockout by CRISPR‐Cas9 Reduces Bladder Cancer Malignancy But Does Not Synergize With BCG and Anti‐PD‐L1 in an Orthotopic Mouse Model

**DOI:** 10.1002/iub.70130

**Published:** 2026-09-07

**Authors:** Juliana Alves de Camargo, Maria Carolina Lee, Milena Monteiro de Souza, Luana Pereira da Silva, Giovanna Vilas Boas Caetano, Karina Serafim da Silva, Carolina Mie Mioshi, Ruan Cesar Aparecido Pimenta, Patrícia Candido, Iran Amorim da Silva, Ericka Barbosa Trarbach, Katia Ramos Moreira Leite, William Carlos Nahas, Sabrina Thalita dos Reis

**Affiliations:** ^1^ Laboratory of Medical Investigation (LIM55), Urology Department University of Sao Paulo Medical School Sao Paulo Brazil; ^2^ Moriah Institute of Science and Education (MISE) Hospital Moriah Sao Paulo Brazil; ^3^ Precision Immunology Institute, Department of Immunology and Immunotherapy, and Tisch Cancer Institute Icahn School of Medicine at Mount Sinai New York New York USA; ^4^ Laboratory of Cellular and Molecular Endocrinology (LIM25), Endocrinology Department Medicine School, University of São Paulo (FMUSP) São Paulo Brazil; ^5^ Uro‐Oncology Group, Urology Department Institute of Cancer State of São Paulo (ICESP) São Paulo Brazil

**Keywords:** BCG, bladder cancer, CRISPR‐Cas9, immunotherapy, MMP9, PD‐L1, tumor microenvironment

## Abstract

Bladder cancer (BC) is the 10th most common cancer worldwide, accounting for approximately 5% of new cases. Several factors contribute to tumor progression, including increased MMP9. Recently, CRISPR‐Cas9 and immunotherapy have offered high specificity for treatments; therefore, we edited MMP9 using CRISPR‐Cas9 methodology to inhibit metastatic mechanisms and evaluated potential synergy with BCG and anti‐PD‐L1 therapy. We performed CRISPR‐Cas9 gene editing using an RNP complex in the MB49 murine BC cell line. Gene expression of MMPs, integrins, and BAX was analyzed, along with protein expression. Cells were divided into a Scramble control group and CRISPR‐Cas9 for the MMP9 edited group (MMP9−/−). Female C57BL/6 mice received orthotopic BC cells (scramble and MMP9−/− groups) treated with BCG and anti‐PD‐L1. Statistical analyses were performed using *t* test or ANOVA. MMP9 gene and protein expression were reduced in the MMP9−/− group compared with the scramble group. No differences were observed in other MMPs. Decreased ITGB3, increased BAX gene expression, as well as reduced migration and adhesion to ECM were observed in the MMP9−/− group. Animals in the scramble group showed greater weight loss and lower survival than treated groups. Overall, MMP9 modulated key cellular mechanisms, but did not show synergistic effects with BCG and anti‐PD‐L1 in vivo.

## 1 Introduction

Bladder cancer (BC) is the tenth most common malignancy worldwide, with approximately 549,393 new cases and 199,922 deaths annually [1, 2]. It is more frequent in men and in patients over the age of 65 [3]. Among the risk factors, smoking stands out as the main risk factor for BC, accounting for approximately 50% of cases [4]. In the most aggressive cases of the disease, treatment is carried out with cisplatin‐based chemotherapy or, alternatively, carboplatin or gemcitabine. However, some patients do not respond to any of these therapies, requiring further studies to understand the cellular and molecular mechanisms of BC [5, 6, 7].

Matrix metalloproteinases (MMPs) are proteins that make up a family of enzymes that degrade various components of the extracellular matrix (ECM). This matrix is present in the structure of solid tumors, providing support to the cells that grow at the site [8]. Several tumors show high expression of these metalloproteinases, mainly MMP2 and MMP9, which are gelatinases that play an important role, including healing, angiogenesis, inflammation, embryogenesis, and carcinogenesis. The expression of most MMPs can be induced by cytokines, growth factors, chemical agents, physical stress, oncogenes, and interactions with the ECM. In BC, increased MMPs expression is correlated with disease progression and recurrence [9]. Therefore, the best way to study the role of metalloproteinases in tumors is to silence or block their expression in cells and evaluate growth, migration, and adhesion capacity. One of the most recent techniques, which have gained prominence for performing gene editing of one or more molecular targets, is the CRISPR‐Cas9 method [10].

Some studies have already demonstrated a significant effect of CRISPR‐Cas9 gene editing in hematological diseases, in which receptors in lymphocytes were blocked and then reintroduced into patients, thereby improving therapeutic response [11].

Today, immunotherapy is used in the treatment of metastatic urothelial cancer. Programmed cell death protein 1 (PD‐1) inhibitors and two programmed cell death ligand 1 (PD‐L1) inhibitors are used in patients who have progressed during or after platinum‐based therapy. Pembrolizumab showed a difference in overall survival. The Food and Drug Administration has approved Atezolizumab and pembrolizumab as inhibitors for patients who are not eligible for cisplatin [12]. Intravesical instillation of BCG (Bacillus Calmette–Guérin) is the standard treatment for patients with noninvasive BC (NMIBC). It prevents high‐grade BC recurrence and can prolong survival by inducing local recruitment of innate and adaptive immune responses [13].

However, the role of MMP9 in modulating tumor behavior and the potential interaction with immunotherapeutic strategies in BC remain incompletely understood. Therefore, this study aimed to investigate the impact of MMP9 knockout using CRISPR‐Cas9 on BC progression and to evaluate its association with BCG and anti‐PD‐L1 therapy.

## 2 Materials and Methods

### 2.1 Cell Culture

The murine BC MB49 cell line was used and cultured in DMEM medium supplemented with 10% fetal bovine serum (FBS) and 1% antibiotic and antifungal solution (Applied Biosystems, Foster city, CA, USA). Cells were incubated at 37°C with 5% CO_2_, and the culture medium was changed every 2 days (Figure S1).

### 2.2 CRISPR‐Cas9

CRISPR‐Cas9 RNP kit (Integrated DNA Technologies, IDT, USA) was used. Initially, we assembled the tracrRNA + crRNA complexes, using the specific sequence for MMP9 (GTTTGGAATCGACCCACGTC) by slow heating and cooling, following the manufacturer's recommendations. Cas9 enzyme was then added to form the RNP complex. The complex was transfected into cells using Lipofectamine RNAiMAX (Applied Biosystems, CA, USA), seeding 3000 cells per well in suspension. Forty‐eight hours after transfection, the culture medium was changed. Experiments were performed in triplicate across independent assays, as described by Zhang et al. [14].

To validate gene editing, conventional PCR was performed directly from the cell pellet, according to the manufacturer's protocol (Integrated DNA Technologies, IDT, USA). Thus, the study was conducted by the Scramble group, which received the kit machinery without the gene‐editing sequence, and the group edited for MMP9 (MMP9−/−).

### 2.3 RNA Isolation and Real‐Time PCR

For gene expression analysis, RNA was extracted from the MB49 strain using the mirVana kit (Ambion, Austin, TX, USA) according to the manufacturer's recommendations. With this kit, total RNA was isolated from the trypsinized cell pellet. The extracted mRNA samples were quantified using the Qubit Quantification Platform (Invitrogen Ltd., Paisley, UK).

After RNA quantification, complementary DNA (cDNA) was synthesized using random primers and the MultiScribe Reverse Transcriptase enzyme (Applied Biosystems, Foster City, CA, USA) according to the manufacturer's recommendations. The samples were subjected to temperature cycles: 10 min at 25°C, 120 min at 37°C, and 5 min at 85°C. After completion of the cycles, the cDNA was stored at −20°C.

Gene expression reactions were performed according to the kit (Applied Biosystems TaqMan Universal PCR Master Mix) for RNA (Table S1). The thermocycler used was ABI 7500 Fast (Applied Biosystems, CA, USA) in standard mode. For sample quantification, TaqMan reagent (Applied Biosystems, CA, USA) was used at the following concentrations: 0.5 μL of specific primer; 5 μL of TaqMan master mix; 2.5 μL of nuclease‐free water; and 2.0 μL of cDNA (concentrated at 15 ng of RNA). Gene expression levels were calculated using the 2^−Δ*Ct*^ method with Applied Biosystems Data Assist Software. All reactions were performed in duplicate, and the β2‐microglobulin (B2M) gene (Applied Biosystems, CA, USA) was used as an endogenous control for RNA.

### 2.4 Western Blotting

To evaluate MMP9 protein activity (92 kDa), Western blotting was performed on samples from the in vitro experiments.

RIPA lysis buffer (Thermo Fisher Scientific, Illinois, USA) was added to the samples, and after shaking, the samples were centrifuged at 11,000 rpm for 20 min at 4°C. All samples were analyzed using both the Qubit Quantification Platform (Invitrogen Ltd., Paisley, UK). After protein quantification, Laemmli buffer was added at a 1:2 ratio. The solution was then subjected to electrophoresis on a 10% polyacrylamide gel. The PageRuler Plus Prestained Protein molecular weight marker (Thermo Fisher Scientific, Illinois, USA) was used. The samples were applied to the polyacrylamide gel at a concentration of 50 μg/well. Next, the combination was submerged in a running buffer. After running, the proteins were transferred to a nitrocellulose membrane submerged in a glycine‐Tris (1×) transfer buffer at 4°C. The membranes were then blocked with 1% BSA in TBS‐T (Tris‐buffered saline Tween 20) for 15 s. Incubation with the antibodies used in the study was performed in the SNAP device (SNAP i.d. system—Millipore), which optimizes incubation times using a vacuum system. For the detection of these membranes, the Luminata Forte substrate (Western HRP Substrate—Millipore) was used, which detects proteins labeled with antibodies. Alliance 4.7 (Uvitec, Cambridge) was used to obtain the images.

### 2.5 Immunofluorescence

The immunofluorescence assay was performed on cells plated in a Nunc Lab‐Tek Chamber Slide System 8‐well plate (Thermo Fisher Scientific, Illinois, USA). After transfection, cells were fixed with 4% paraformaldehyde in PBS for 10 min, followed by three washes with 1× PBS for 5 min each. Next, blocking was performed with 3% BSA for 30 min. Subsequently, the cells were incubated overnight with the primary antibody (anti‐MMP9 mouse monoclonal antibody, MA5‐15886; or anti‐Cas9 mouse monoclonal antibody 7A9‐3A3; Thermo Fisher Scientific, Illinois, USA) in 1% BSA solution in PBS, at a concentration of 1:300. The next day, the cells were washed with PBS three times for 5 min and incubated with the secondary antibody Alexa Fluor 647 AffiniPure goat antimouse (Jackson ImmunoResearch 115‐605‐003) at 1:200 (red color) for 2 h. After the last wash with PBS, the cells were marked with ProLong Gold Antifade Mountant for nuclei staining with DAPI (Thermo Fisher Scientific, Illinois, USA). The images were captured on a Leica TCS SP2 II laser‐scanning microscope (20× magnification).

### 2.6 Migration Assay

MB49 cells were cultured in 24‐well plates containing untransfected cells (control group) and transfected cells (MMP9−/−). The experiments were performed in triplicate. After 24 h, a straight line was drawn with a sterile 200 μL micropipette tip to scratch the cell layer in each well. The distance between cells was measured and photographed at 0, 24, and 48 h. For each image (20×), the distance was measured using NIS‐Elements D 3.1 (Nikon). The migration distance was calculated as a percentage using the formula: (Initial D—Final D)/(Initial D) × 100 = % migration, where D is the distance between the edges of the scratch [15].

### 2.7 Apoptosis and Proliferation Assays by Flow Cytometry

After the transfections, cellular proliferation and apoptosis were analyzed using the Muse Cell Analyzer (Merck Millipore, Burlington, MA, USA). The cells were labeled with the Muse Ki67 Proliferation kit (MCH100114) and the Muse Annexin V & Cell Death kit (MCH100105), according to the manufacturer's instructions.

### 2.8 Decellularization of the Bladder andRecellularization of the ECM With the MB49 Cell Line

Porcine bladder tissue was used for decellularization following approval by the Institutional Animal Care and Use Committee (protocol no. 2380/2025). The bladder was dissected between the urothelium and detrusor muscle. Bladder decellularization was performed with 1% SDS under agitation for 24 h at room temperature, with the solution being changed every 3 h. After this process, the tissues were submerged in a 1% Triton solution for 6 h at 4°C. Next, the ECM was submerged in milliQ water for 24 h at room temperature, with 10% antibiotic and changes every 3 h. The decellularized ECM was stored in PBS with 10% antibiotic. After decellularization, we placed the ECM, which had been soaked in culture medium, in a CO_2_ incubator at 37°C for 2 h. We changed the culture medium and added 50,000 MB49 cells on top of the ECM using only 15 μL of culture medium, then placed in the incubator for another 2 h. We completed the plate with the culture medium at the ideal volume and finished the experiment 7 days after recellularization.

### 2.9 In Vivo Experiments

Female C57BL/6 mice were used following approval by the Institutional Animal Care and Use Committee (CEUA, protocol no. 1710/2021). Animals were anesthetized with 2.0%–3.0% isoflurane (induction) and 1.5%–2.0% (maintenance). The lower abdomen and urethral meatus were disinfected with 2% chlorhexidine gluconate. The urethral meatus was catheterized with a 24G Jelco catheter (BD Insyte Autoguard, BD Biosciences; #38181214) and the bladder was emptied. The bladder mucosa was then prepared by instilling 20 μL of 0.25% intravesical trypsin (Thermo Fisher Scientific, Illinois, USA) for 15 min. After draining the trypsin, the bladder was irrigated with FBS to inactivate the trypsin, and then 50 μL of the cell suspension was instilled into the bladder. Cells were cultured in DMEM medium supplemented with 10% FBS and 1% antibiotic and antimycotic solution (Thermo Fisher Scientific, Illinois, USA). The catheter was removed, and the urethral meatus was occluded using a microsurgical autostatic vascular clamp (Vascu‐statt midi, Scanlan; #1001–500). The solution remained intravesical for 45 min, with the animal kept under anesthesia. After this period, the clamp was removed and the bladder was emptied. Animals were divided into four groups: Group 1: orthotopic grafting of MB49 cells from the Scramble group; Group 2: Scramble + intravesical BCG treatment + intraperitoneal anti‐PD‐L1 treatment; Group 3: CRISPR‐Cas9‐edited cells for MMP9 (MMP9−/−); Group 4: MMP9−/− + intravesical BCG treatment + intraperitoneal anti‐PD‐L1 treatment. A sample size calculation was performed using changes in the animals' body weight as the primary outcome measure. We used eight animals per group [16]. Five days after inoculation, the animals received their respective treatments, with BCG administered weekly at 2 × 10^6^ CFU in a 50 μL solution diluted in PBS until the end of the experiment [17, 18]. (UROHIPE— *Mycobacterium bovis* —40 mg/mL; FA #1132G039). The anti‐PD‐L1 antibody (BioXcell, BE0083) was administered three times per week at 10 mg/kg [7, 19]. Animals were monitored daily for 15 days. After this period, animals were euthanized, and bladders were collected, fixed in 10% formalin and processed routinely. The slides were stained with Hematoxilin and eosin and were examined by a uropathologist (KRML). The analysis was based on standard tissue analysis criteria. Histological evaluations focused on cross‐sections at 200× magnification.

### 2.10 Statistical Analysis

The statistical analyses were performed using GraphPad Prism 9.0 (GraphPad Software, CA, USA). The Shapiro–Wilk test was used to assess the normality of the data. Student's *t* test or ANOVA was used for comparisons with two and four groups respectively. Survival analysis was performed using the Kaplan–Meier estimator, and the curves were compared using the Log‐rank (Mantel‐Cox) test. The level of statistical significance was set at 5% (*p* ≤ 0.05).

## 3 Results

### 3.1 Gene Editing With CRISPR‐Cas9 for MMP9 in the MB49 Cell Line

**FIGURE 1:**
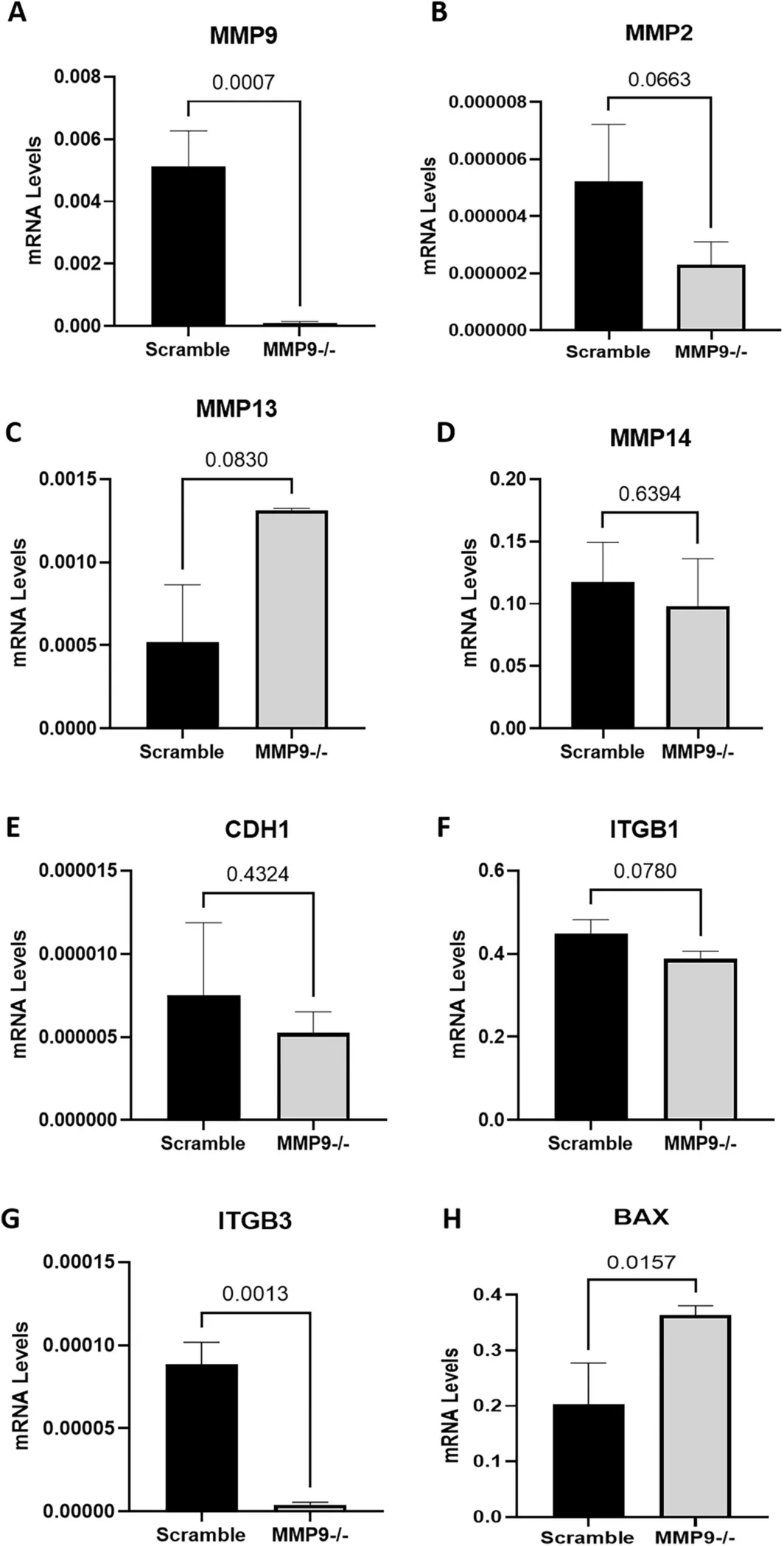
Gene expression of metalloproteinases, integrins, and apoptosis in the MB49 murine BC cell line from the Scramble and CRISPR‐Cas9‐edited MMP9 cells (MMP9−/−). (A) MMP9 gene expression in samples from the control group (Scramble) and the group edited with CRISPR‐Cas9 for MMP9 (MMP9−/−). (B) MMP2 gene expression in samples from the scramble and MMP9−/− groups. (C) MMP13 gene expression in samples from the scramble group and the MMP9−/− group. (D) MMP14 gene expression in samples from the scramble group and the MMP9−/− group. (E) CDH1 gene expression in samples from the scramble group and the MMP9−/− group. (F) ITGB1 gene expression in samples from the scramble group and the MMP9−/− group. (G) ITGB3 gene expression in samples from the scramble group and the MMP9−/− group. (H) BAX gene expression in samples from the scramble group and the MMP9−/− group. Statistical significance set at *p* < 0.05.

After transfecting the CRISPR‐Cas9 system for MMP9, we observed a significant decrease in gene expression in the edited cells (MMP9−/−) compared to the Scramble group (*p* = 0.0007) (Figure 1A). We also evaluated the gene expression of other metalloproteinases, such as MMP2, MMP13, and MMP14, and observed no significant difference in gene expression (Figure 1B–D). In addition, we evaluated integrin expression (CDH1, ITGB1, and ITGB3) and observed a statistical difference only for ITGB3, with decreased gene expression in the MMP9−/− group compared to the Scramble group (*p* = 0.0013) (Figure 1E–G). When evaluating the gene expression of BAX, we observed an increase in expression in the MMP9−/− group compared to the Scramble group (*p* = 0.0157) (Figure 1H).

To validate the data, we confirmed the editing using GFP fluorescence, conventional PCR, and fragment digestion with T7 endonuclease I. In the edited MMP9−/− samples, we observed PCR and digestion bands that were absent in the Scramble group (Figure S2).

**FIGURE 2:**
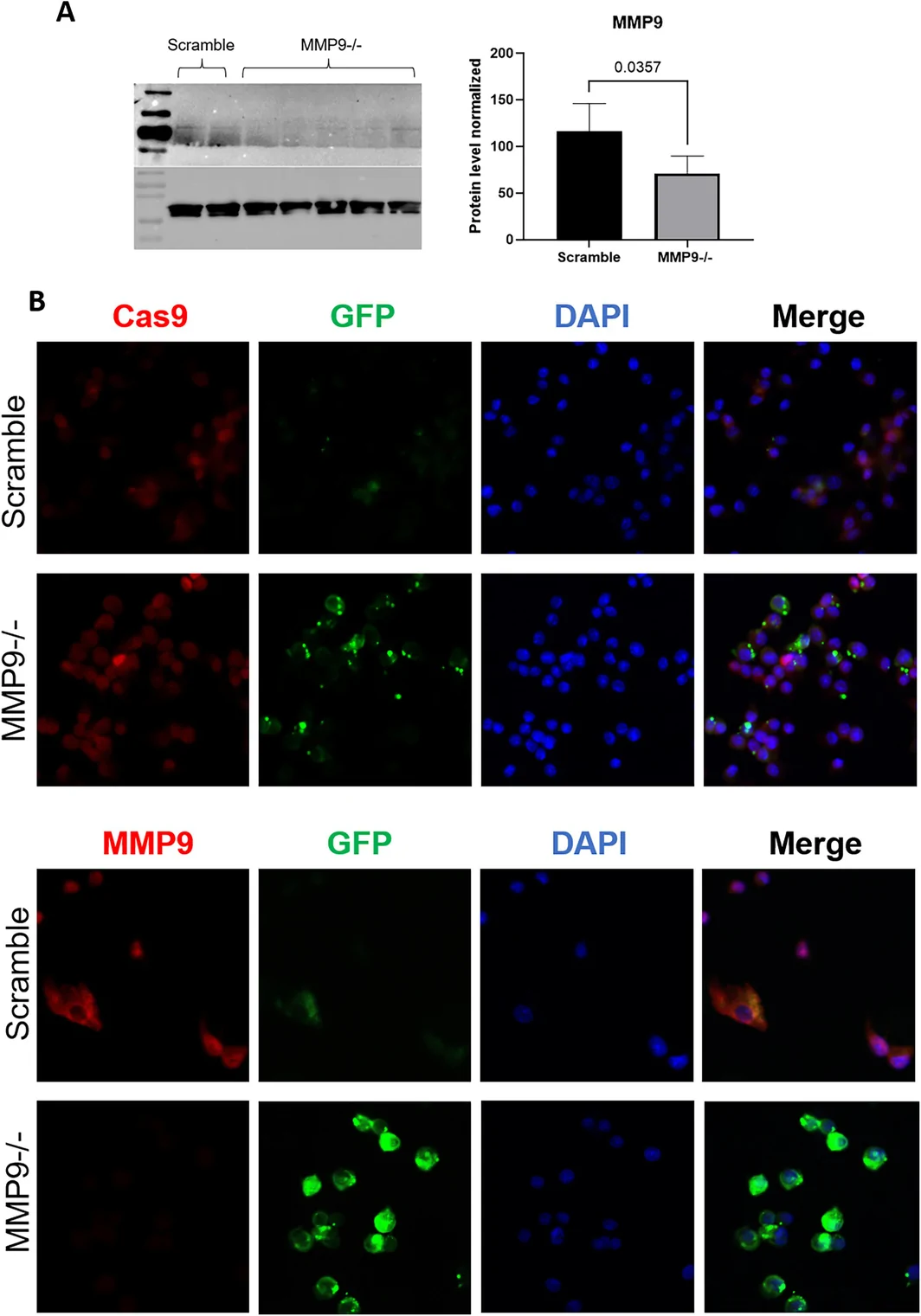
Analysis of MMP9, Cas9, and GFP protein expression in the MB49 murine BC cell line from the Scramble and MMP9‐edited cells with CRISPR‐Cas9 (MMP9−/−) groups. (A) Western blotting of samples from the scramble and MMP9−/− groups. (B) Immunofluorescence with red (Cas9 and MMP9), green (GFP), and blue (DAPI) nuclear staining in the scramble and MMP9−/− groups. Statistical significance set at *p* < 0.05.

To validate the results, we performed western blotting and observed a decrease in MMP9 protein expression in the MMP9−/− cell group compared to the Scramble group (*p* = 0.0357) (Figure 2A). In addition, in immunofluorescence, we validated the results by confirming the presence of Cas9 protein and, in both the Scramble and MMP9−/− groups, the presence of GFP and the absence of MMP9 in the edited group (MMP9−/−) compared to the Scramble group (Figure 2B).

**FIGURE 3:**
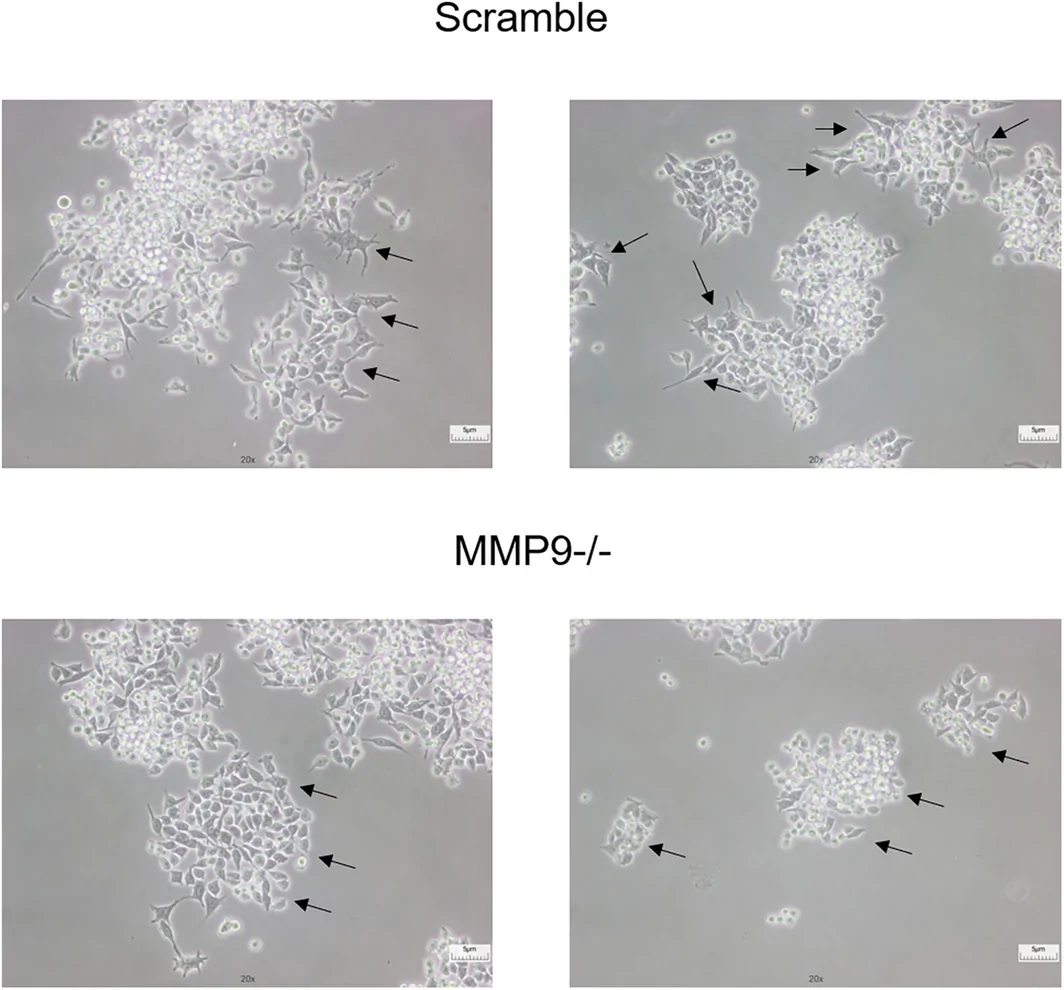
Cell morphology in the MB49 murine BC cell line from the Scramble and MMP9‐edited cells with CRISPR‐Cas9 (MMP9−/−) groups.

We performed a qualitative morphological analysis an optical microscope of cells in the MMP9−/− group compared with the Scramble group. We observed minor changes in cell membranes and possibly in cell–cell interactions, with the Scramble group showing a more irregular cell shape. The MMP9−/− group, on the other hand, presented smaller and more closed cells (Figure 3).

**FIGURE 4:**
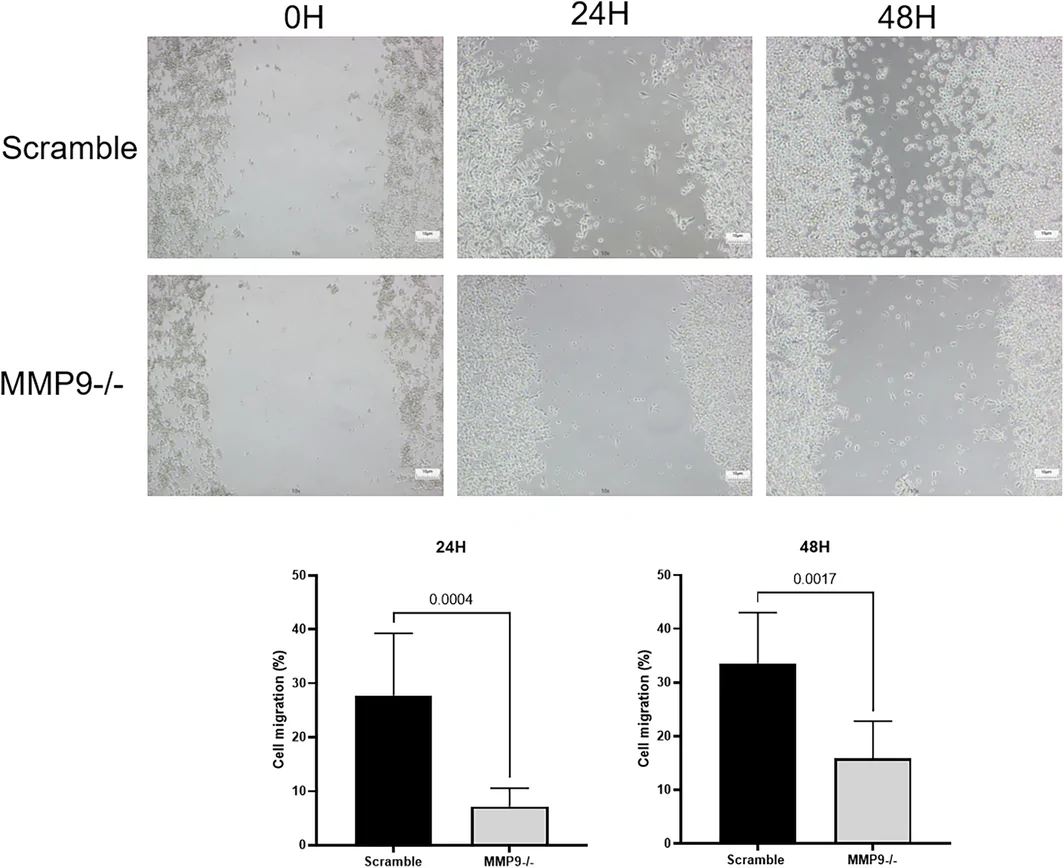
Cell migration at 0, 24, and 48 h in the MB49 murine BC cell line from the Scramble and MMP9‐edited cells with CRISPR‐Cas9 (MMP9−/−) groups. Statistical significance set at *p* < 0.05.

To evaluate the role of MMP9 in cell migration capacity, we performed a migration assay. MMP9−/− cells showed a significantly reduced migration percentage compared with the Scramble group at both 24 h (*p* = 0.0004) and 48 h (*p* = 0.0017). At 24 h, migration was approximately 10% in the MMP9−/− group versus 35% in the scramble group; at 48 h, migration was approximately 25% versus 40%, respectively (Figure 4).

**FIGURE 5:**
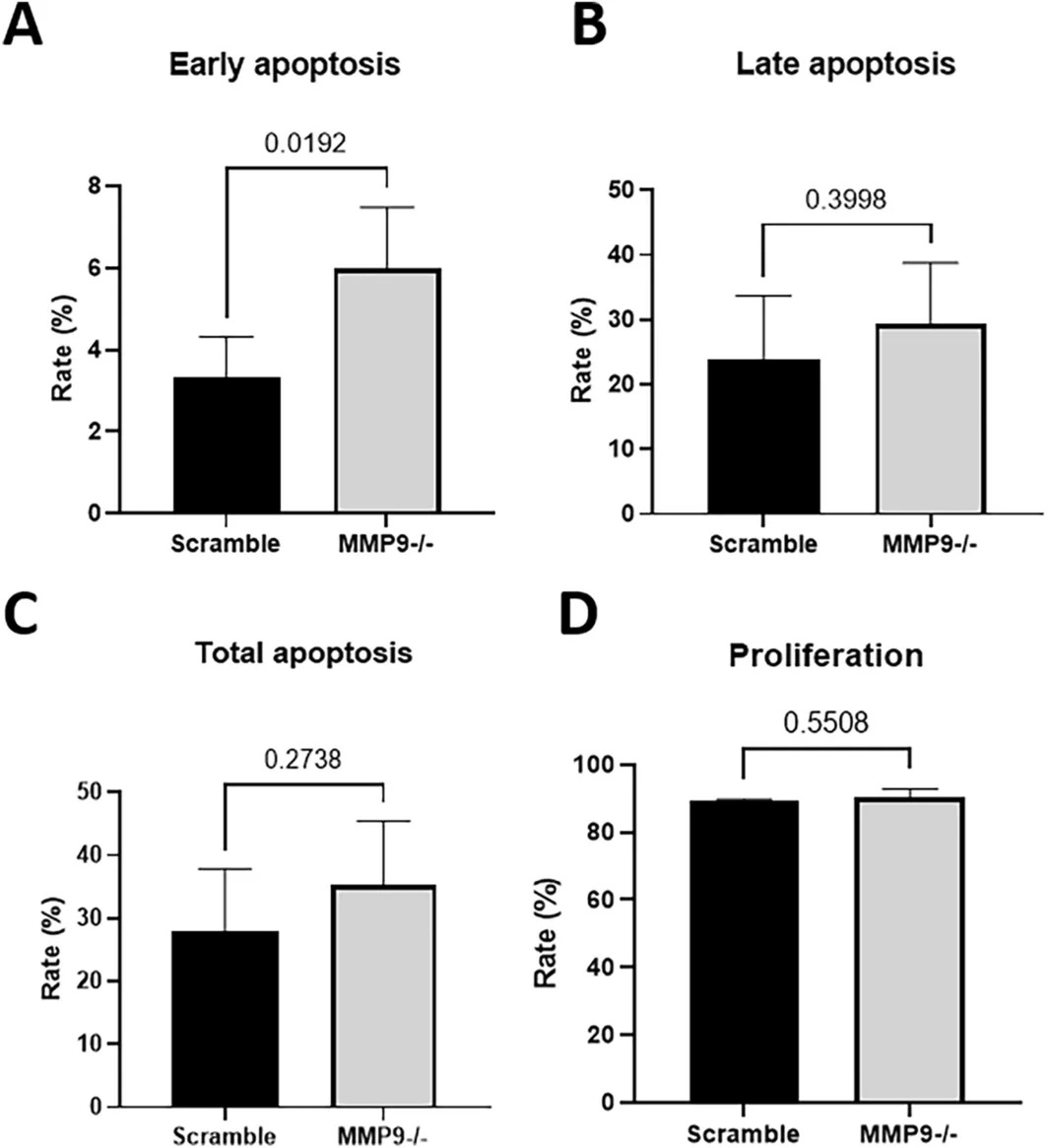
Apoptosis and cell proliferation by flow cytometry in the MB49 murine BC cell line from the Scramble groups and cells edited for MMP9 with CRISPR‐Cas9 (MMP9−/−). (A) Early apoptosis in the Scramble and MMP9−/− groups. (B) Late apoptosis in the Scramble and MMP9−/− groups. (C) Total apoptosis in the Scramble and MMP9−/− groups. (D) Proliferation in the Scramble and MMP9−/− groups. Statistical significance set at *p* < 0.05.

In addition, we evaluated cell apoptosis by flow cytometry and found that the MMP9−/− group had a higher early apoptosis rate than the Scramble group (*p* = 0.0192) (Figure 5A). However, we observed no difference between the groups in late or total apoptosis rates (Figure 5B,C). Regarding cell proliferation, no difference was observed between the groups studied (Figure 5D).

**FIGURE 6:**
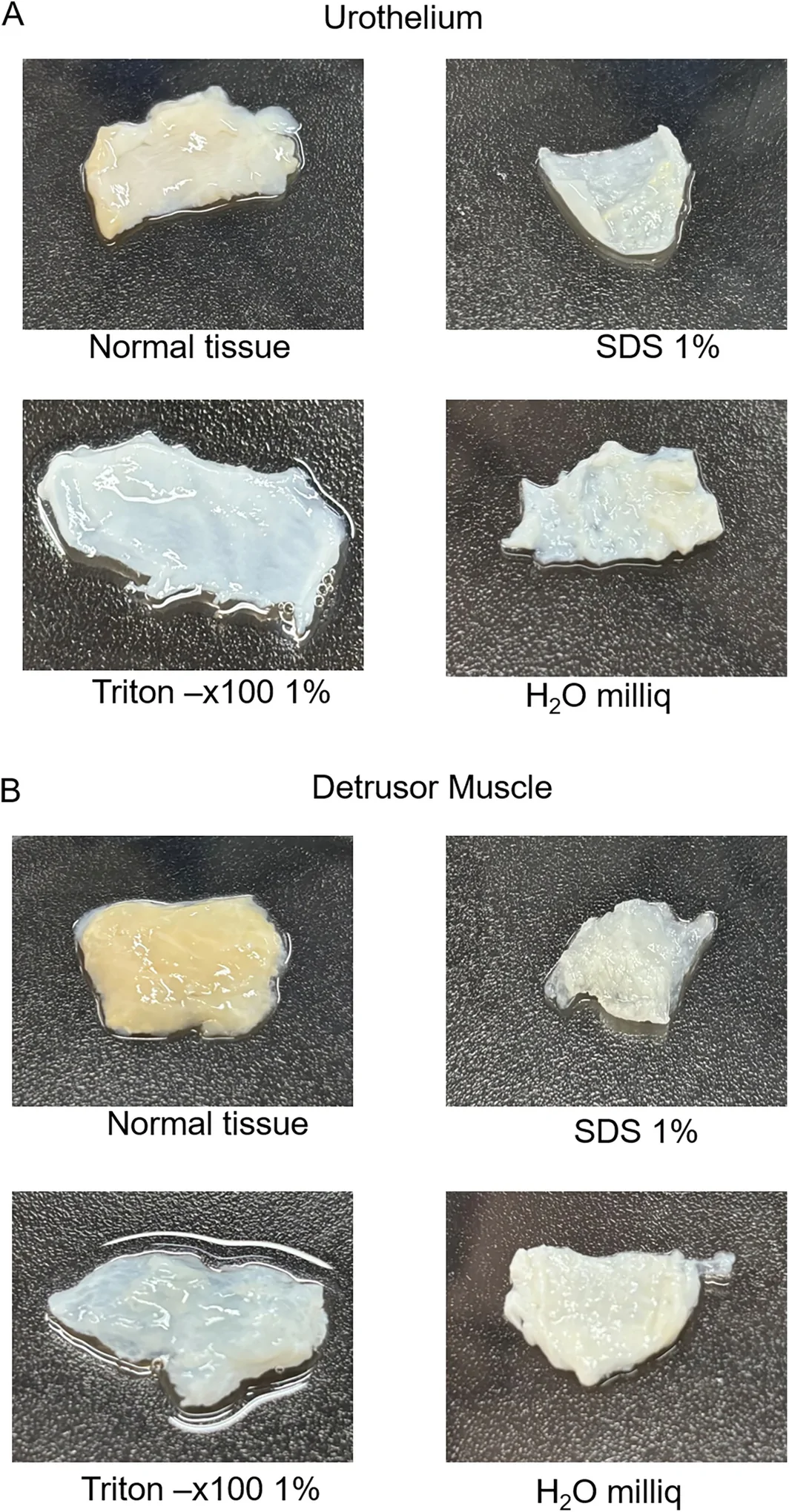
Dissection of porcine bladders into urothelium and detrusor muscle and tissue decellularization to obtain the extracellular matrix (ECM). (A) Decellularization of urothelium with 1% SDS in 24 h; 1% Triton‐X100 in 6 h, and H2O for 24 h. (B) Decellularization of the detrusor muscle with 1% SDS in 24 h; Triton‐X100 1% in 6 h, and H2O for 24 h.

To evaluate the potential for cells to adhere to ECM derived from the tissue of origin, we performed bladder decellularization assays. We successfully standardized the protocol in our laboratory, aiming to remove tissue cells while preserving ECM structural proteins. The layers of the porcine bladder were separated into urothelium (Figure 6A) and detrusor muscle (Figure 6B), and decellularization was performed with detergents and preservation of the ECM.

**FIGURE 7:**
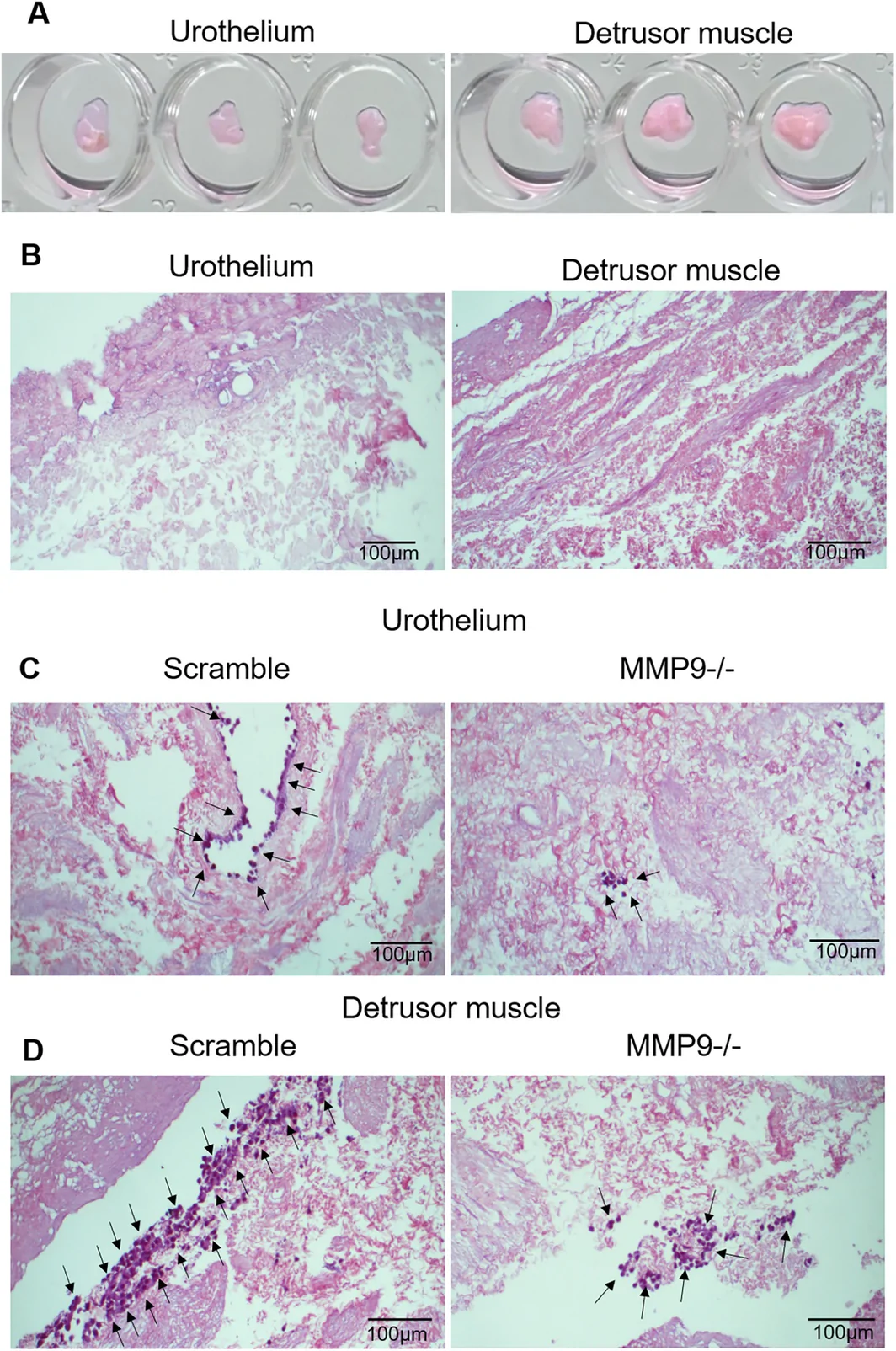
Recellularization of ECM with the MB49 murine BC cell line from the Scramble and MMP9‐edited cells with CRISPR‐Cas9 (MMP9−/−) groups and histopathological analysis. (A) ECM of the urothelium and detrusor muscle in a 24‐well culture plate. (B) Histological analysis of the ECM of the urothelium and detrusor muscle. (C) Recellularization of urothelium ECM with the Scramble and MMP9−/− groups. (D) Recellularization of detrusor muscle ECM with the Scramble and MMP9−/− groups.

After this process, the cells were plated (Figure 7A). When evaluating the histopathology of the ECM, we observed the absence of tissue cells and nuclei in both the urothelium and detrusor muscle (Figure 7B). We then evaluated the cell adhesion potential of the MB49 lineage to the ECM in both the urothelium and detrusor muscle. In the ECM where the Scramble group cells were plated, we observed a higher number of cells than in the MMP9−/− group (Figure 7C,D).

### 3.2 In Vivo Studies

**FIGURE 8:**
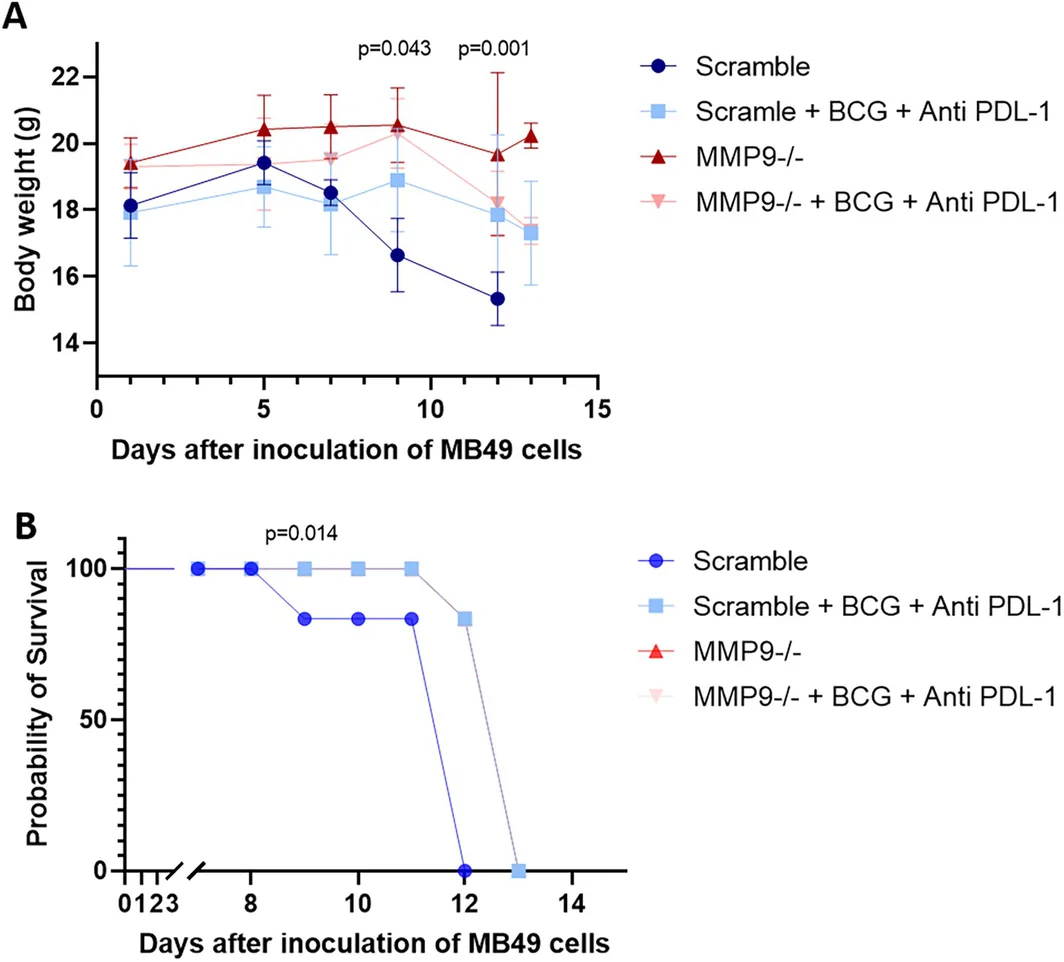
In vivo experiments: Treatment with BCG and anti‐PD‐L1 in animals with orthotopic grafting of the MB49 murine BC cell line from the Scramble and MMP9−/− groups. (A) Body weight of female C57BL/6 mice three times a week for 15 days of experimentation, divided into four groups: Scramble; Scramble + BCG + anti‐PD‐L1; MMP9−/−; MMP9−/− + BCG + anti‐PD‐L1. (B) Survival curve of female C57BL/6 mice during 15 days of experiment, divided into four groups: Scramble; Scramble + BCG + anti‐PD‐L1; MMP9−/−; MMP9−/− + BCG + anti‐PD‐L1. Statistical significance set at *p* < 0.05; *N* = 8 per group.

We inoculated the cells into animals and observed that the Scramble group showed significant weight loss compared to the other groups that received the treatments (*p* = 0.043) (Figure 8A). In addition, we also evaluated survival and observed that the Scramble group had a lower rate compared to the treated groups (*p* = 0.014) (Figure 8B).

**FIGURE 9:**
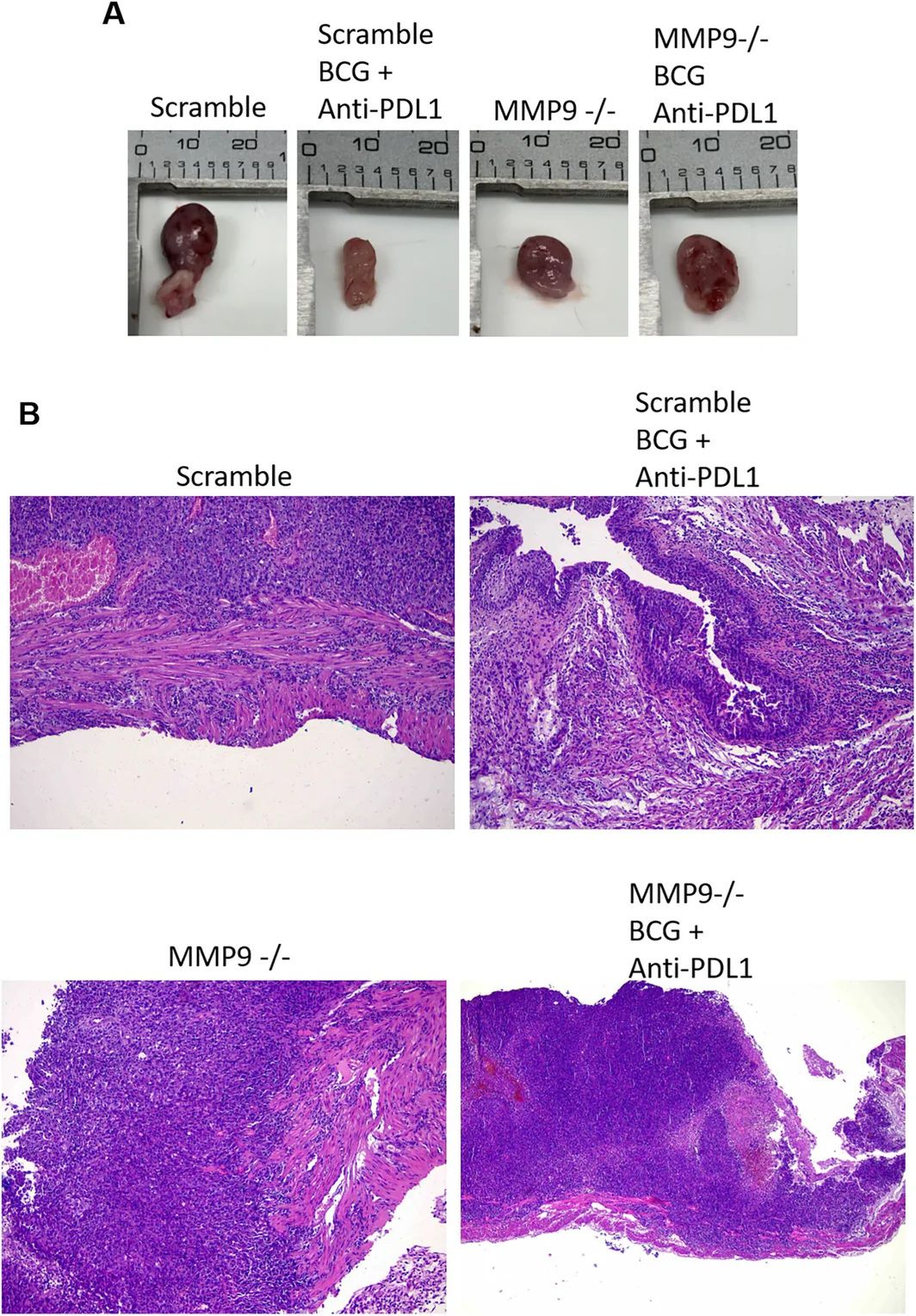
Macroscopic and histopathological parameters of animals with orthotopic grafts of the MB49 murine BC cell line from the Scramble and MMP9−/− groups. (A) Images of the bladder with internal tumors in female C57BL/6 mice during 15 days of experimentation, divided into four groups: Scramble; Scramble + BCG + anti‐PD‐L1; MMP9−/−; MMP9−/− + BCG + anti‐PD‐L1. (B) Images of histopathological analysis of female C57BL/6 mice during 15 days of experiment, divided into four groups: Scramble; Scramble + BCG + anti‐PD‐L1; MMP9−/−; MMP9−/− + BCG + anti‐PD‐L1.

Macroscopic tumor analysis revealed that the Scramble group had greater bleeding and a larger tumor inside the bladder compared with the other groups. All animals developed tumors, but treated animals presented less bleeding and reduced vessel formation compared to the untreated Scramble group (Figure 9A).

Histological analysis (H&E staining) revealed high‐grade, aggressive, and invasive urothelial tumors. Scramble group treated with BCG + anti‐PD‐L1 was less aggressive than the untreated Scramble group. The other two groups, MMP9−/− and MMP9−/− + BCG + anti‐PD‐L1, presented aggressive tumors (Figure 9B).

## 4 Discussion

BC remains a highly prevalent malignancy, particularly in advanced stages where treatment is associated with substantial morbidity [20, 21]. In this study, we evaluated the roles of MMP9, conventional BCG, and anti‐PD‐L1 treatments in an invasive muscle BC cell line. In the cell line, we standardized MMP9 blockade using the CRISPR‐Cas9 methodology in vitro and validated it by qPCR, conventional PCR, western blotting, and immunofluorescence (Figures 1 and 2, and Supporting Information). Our data show low levels of gene and protein expression, as determined by qPCR and Western blotting, respectively. We attribute these findings to residual nonfunctional RNA and nonspecific antibody binding. This interpretation is supported by the absence of MMP9 expression detected via immunofluorescence in the CRISPR‐Cas9‐edited group compared with the scramble control group. Immunofluorescence, which allows the assessment of protein expression at the single‐cell level, is considered the most precise technique for this purpose. Currently, several studies have employed this methodology to evaluate molecular markers in vitro. These data indicated that the validations performed in our study are consistent with descriptions in the literature [22, 23, 24]. Therefore, we evaluated the influence of MMP9 gene editing on the expression of other metalloproteinases, including MMP2, MMP13, and MMP14 (Figure 1), and observed no significant differences in the expression between groups. This result, coupled with the manufacturer's certified quality assurance for the kit, highlights the high efficiency and precision of MMP9 gene editing, suggesting the absence of possible off‐targets. In the literature, most studies do not investigate the impact of gene editing on members of the same marker family or potential off‐target effects, underscoring the relevance of our analysis.

Postgene editing, optical microscopic evaluation of cell behavior and proliferation revealed that control group cells exhibited aberrant cell membrane morphology, characterized by irregular contours, possibly related to cell–cell connections. In contrast, this characteristic was not clearly observed in the MMP9‐edited cells (Figure 3). These findings suggest that gene editing can modulate cellular morphological aspects. Our results are consistent with previously published data from our group, which showed similar morphological changes in prostate cancer cell lines edited with CRISPR‐Cas9 targeting MMP9 [25]. In addition, we evaluated integrin gene expression (CDH1, ITGB1, and ITGB3) and observed reduced ITGB3 expression in MMP9‐edited cells compared with the control group. The literature indicates that increased integrin expression in some tumor models, including BC, may be associated with a worse prognosis [26]. Thus, the reduction in ITGB3 gene expression observed here may reflect a less aggressive cellular phenotype (Figure 1).

Next, we evaluated cell migration and found that MMP9‐edited cells had reduced migratory potential compared to the control group, consistent with the literature (Figure 4). Pan et al. demonstrated that MMP9 silencing in gliomas was associated with reduced cell migration [27]. Similarly, Nalla et al. observed that MMP9 inhibition in prostate cancer reduces cell migration and invasion [28].

We evaluated the apoptosis and proliferation, showing an increased early apoptosis in MMP9‐edited cells, with no significant differences in late apoptosis, total apoptosis, or cell proliferation compared to the Scramble group [29].

We also decided to investigate the potential for cell adhesion to ECM obtained from urothelium and detrusor muscle [30, 31]. We adapted and standardized this methodology to recellularize the ECM of the urothelium and detrusor muscle using the MB49 cell line to evaluate their adhesion capacity. This method is more effective than conventional cellular seeding with Matrigel because we used ECM derived from the bladder, and thus were able to assess the behavior of BC cells in this context. The results showed a cell pattern similar to high‐grade carcinoma in situ (Figure 7). We observed that ECM recellularized with MMP9‐edited cells had fewer adherent cells than the ECM with scramble cells, both in the urothelium and muscle (Figures 6 and 7). These results indicate that gene editing can reduce the potential for cell adhesion in three‐dimensional cultures. Previous studies have also explored bladder decellularization beyond regenerative purposes, supporting the relevance of this model [32].

In the in vivo experiments, the control group showed greater weight loss and lower survival than the treated groups. However, no significant differences were observed among the treatment groups (Figures 8 and 9). The group that received MMP9‐edited cells showed lower weight loss and higher survival, but similar results were observed in the group treated with BCG and anti‐PD‐L1. These findings suggest that both interventions had beneficial effects; however, no synergy was observed in animals receiving the triple combination (MMP9‐edited + BCG + anti‐PD‐L1). Thus, we believe that the treatments had isolated effects, but the synergy did not potentiate the results. Previous studies have reported that BCG, alone or in combination with other treatments, has beneficial effects in NMIBC [33, 34, 35]. Other studies using BCG in an orthotopic model reported similar results [36]. In this study, using an orthotopic murine model of BC, we extended previous findings regarding the biological effects of MMP9 gene editing [25, 29]. Overall, our results *suggest a functional link between MMP9 and tumor* cell migration, adhesion, and early apoptotic responses. Although MMP9 blockade did not potentiate the effects of BCG and anti‐PD‐L1 therapy in vivo. In orthotopic model, we were unable to measure the tumors during the experiment, which is a limitation of the study. The histopathological analysis by the uropathologist (KRML) after the experiment opened the door to future studies aimed at improving combinatorial strategies based on gene editing and immunotherapy.

## 5 Conclusions

The gene‐editing methodology using CRISPR‐Cas9 targeting MMP9 proved effective and reproducible, resulting in a significant reduction in gene expression and, more importantly, MMP9 protein expression.

MMP9 editing resulted in increased apoptosis, evidenced by higher BAX gene expression and early apoptosis, and reduced cell migration and adhesion to the ECM, suggesting that MMP9 plays an important role in maintaining tumor cell morphology and invasive behavior.

In in vivo experimental models, the treatments exerted effects, but no additional benefit was observed following the addition of MMP9 suppression to the combined BCG plus anti‐PD‐L1 treatment regimen.

## Funding

This work was supported by the Fundação de Amparo à Pesquisa do Estado de São Paulo (2022/08864‐3, 2022/02288‐0) and the Coordenação de Aperfeiçoamento de Pessoal de Nível Superior.

## Ethics Statement

This study was submitted and approved by the Research Ethics Committee of the University of Sao Paulo Medical School under the number #1710/2021.

## Consent

The authors have nothing to report.

## Conflicts of Interest

The authors declare no conflicts of interest.

## Supporting information


**Figure S1:** MB49 cell line certification.

**Figure S2:** Conventional PCR of scramble samples (C1 and C2), samples edited with CRISPR‐Cas9 for MMP9 (MMP9 1, MMP9 2, MMP9 3), and positive and negative reaction controls.

**Table S1:** Primers used in the study.

## Data Availability

The data that support the findings of this study are available from the corresponding author upon reasonable request.
