# Supplementary material for: MMP9 Knockout by CRISPR‐Cas9 Reduces Bladder Cancer Malignancy But Does Not Synergize With BCG and Anti‐PD‐L1 in an Orthotopic Mouse Model
Source: IUBMB Life. 2026 Sep 7;78(9):e70130. doi: 10.1002/iub.70130 (PMC13550818; doi:10.1002/iub.70130)
Supplement: Supplementary file 1 [file IUB-78-0-s001.docx]

**Supplementary figure 2.** Conventional PCR of scramble samples (C1 and C2), samples edited with CRISPR-Cas9 for MMP9 (MMP9 1; MMP9 2; MMP9 3), and positive and negative reaction controls.
