# Supplementary material for: MMP9 Knockout by CRISPR‐Cas9 Reduces Bladder Cancer Malignancy But Does Not Synergize With BCG and Anti‐PD‐L1 in an Orthotopic Mouse Model
Source: IUBMB Life. 2026 Sep 7;78(9):e70130. doi: 10.1002/iub.70130 (PMC13550818; doi:10.1002/iub.70130)
Supplement: Supplementary file 3 [file IUB-78-0-s003.docx]

**Supplementary table1.** Primers used in the study.

| **Genes** | **Assays** | **Company** |
| --- | --- | --- |
| MMP9 | Mm00442991_m1 FAM | Applied Biosystems |
| MMP2 | Mm00439498_m1 FAM | Applied Biosystems |
| MMP13 | Mm00439491_m1 FAM | Applied Biosystems |
| MMP14 | Mm00485054_m1 FAM | Applied Biosystems |
| BAX | Mm00432051_m1 FAM | Applied Biosystems |
| PDL-1 | Mm03048248_m1 FAM | Applied Biosystems |
| ITGB1 | Mm01253230_m1 FAM | Applied Biosystems |
| GB3 | Mm00443980_m1 FAM | Applied Biosystems |
| CDH1 | Mm01247357_m1 FAM | Applied Biosystems |
| B2M | Mm00437762_m1 VIC | Applied Biosystems |
